# Knowledge sharing practices among African health sciences librarians

**DOI:** 10.5195/jmla.2021.1183

**Published:** 2021-10-01

**Authors:** Olalekan Moses Olayemi, Kemi Jummai Olayemi

**Affiliations:** 1 lekus2000@yahoo.com, Senior Librarian, Library and Information Technology Department, Nigerian Institute of Medical Research, Lagos, Nigeria; 2 prettykem1006@yahoo.com, Librarian, Bayero University Kano, Kano, Nigeria

**Keywords:** knowledge sharing, social exchange theory, librarian, information, AHILA

## Abstract

**Objective:**

The objective of this study was to investigate knowledge sharing practices among health sciences librarians in African countries.

**Methods:**

A cross-sectional survey design was employed. The study population consisted of African health sciences librarians that attended the 16^th^ Biennial Conference of the Association for Health Information and Libraries in Africa on October 14–18, 2019, at the University of Ibadan in Nigeria. Data were collected using a questionnaire and analyzed using descriptive statistics.

**Results:**

The types of knowledge most commonly shared by respondents were information on conferences, workshops, and seminars as well as information on new trends and technologies in librarianship. The main avenue of knowledge sharing was face-to-face interaction. Unwillingness to share knowledge and a lack of awareness about current trends and issues were the top identified challenges to knowledge sharing.

**Conclusion:**

These survey results establish the existence of a low level of knowledge sharing among health science librarians in Africa and suggest that concerted efforts should be made to overcome barriers to knowledge sharing within and across African countries.

## INTRODUCTION

Knowledge is crucial for the development and competitive advantage of individuals and organizations. Knowledge can be considered as ideas gained through learning that are stored in a human brain or encoded in organizational documents, products, facilities, and concepts [1]. Here, we define knowledge as a set of insights, experiences, values, and skills embedded in an individual. However, knowledge—whether tacit or explicit—can only be useful when it is carefully harnessed and shared among individuals, colleagues, groups, organizations, or collaborative parties [2]. According to Yu, Wilkins, and Ma [3], knowledge sharing is a process through which people transmit their knowledge interactively so that individual knowledge turns into organizational knowledge, allowing the acquisition of new skills, experiences, and insights to enhance job output. Furthermore, Lin [4] defines knowledge sharing in terms of its benefits, including helping communities and groups of people with similar objectives work together, facilitating the exchange of knowledge to enhance organizational learning capacity, and increasing the likelihood of achieving organizational and individual goals.

To provide library users with relevant and high-quality information, health sciences librarians rely in part on sharing knowledge with their colleagues [5]. In particular, the exponential growth of medical information over the past decades as a result of continual research, innovation, and application of technology has led to drastic changes in the information-seeking behavior of physicians, patients, and library users, making it increasingly difficult for health sciences librarians to anticipate and meet their users' information needs. As such, Akparobore [6] asserts that knowledge sharing among academic librarians is one of the most convenient ways of reaching a desirable level of knowledge on current trends. Likewise, knowledge sharing has been identified as one of the most effective methods of increasing specialized knowledge among librarians for effective service delivery [7].

This study is anchored in social exchange theory (SET), which was proposed by Peter Blau in 1964. The central point of SET is that people engage in social exchange based on the personal gain derived or derivable from contributing to others' wellbeing. This theory emphasizes interpersonal interaction among individuals as they conduct their activities and exchange valuable information or resources with each other. In the context of the present study, we assume that individuals interact with their professional colleagues for the purposes of self-interest and mutual benefits. As such, knowledge sharing has a tendency to contribute to one's own knowledge, awareness, and skills and thereby enhance job performance while also forming long-term relationships between individuals [8]. Akparobore [6] also reinforces that knowledge sharing among librarians enhances their ability to seek study-related help from one another, facilitates the outcomes of collective learning, and allows individuals to gain from the experiences and practices of others.

Several studies on knowledge sharing have been conducted among librarians and information professionals. Akparobore [6] examined knowledge sharing among librarians in university libraries in the South-South zone of Nigeria and found that although librarians exchanged information and expertise, the practice of knowledge sharing among librarians was quite low. In addition, librarians preferred to share knowledge about information and communication technology (ICT) and networking. Mayekiso [9] found that the benefits of knowledge sharing within academic libraries include learning, staying abreast of new developments within one's institution, and having better-informed staff, which in turn leads to better service delivery.

Different categories of knowledge sharing across media platforms include print-based media (e.g., journals, conference proceeding materials, posters) and computer- or web-based media (e.g., social media, blogs, webinars, online forums, email). New technologies provide new opportunities for libraries and librarians around the world, especially in the areas of services delivery and interactions with professional colleagues. Ogunmodede and Popoola [10] surveyed librarians in federal universities in Nigeria and found that their primary mediums of knowledge sharing were face-to-face interaction, mobile phones, and email. In a study by Quadri and Garaba [11], respondents reported that ICT facilitates new organizational forms of knowledge sharing and enhances the quick delivery and dissemination of knowledge among librarians. Islam and Tsuji [12] investigated informational professionals' perceptions of knowledge sharing practices on social media platforms and found that most respondents were aware of the use of social media for knowledge sharing and were inspired to use social media for its speed and ease of use, management of personal information, ability to allow better contact with users and colleagues, and function as a powerful communication tool. Concerning challenges to information and knowledge sharing, a comparative study by Fari and Ocholla [13] found that academics in select universities in Nigeria and South Africa showed negative attitudes toward knowledge sharing, which they attributed to inadequate sharing support systems. Similarly, a study by Awodoyin and colleagues [14] revealed that major challenges to effective knowledge sharing by librarians were a lack of understanding of how to effectively share knowledge, lack of social networking skills, inability to use modern technology, and failure to appreciate the value of sharing knowledge.

While there is widespread recognition of knowledge sharing among different types of professionals, there are no studies of knowledge sharing among health sciences librarians in Africa. Moreover, Muchaonyerwa [15] and Olatokun and Njideaka [7] point out that knowledge sharing among librarians in Africa is limited. Thus, this study was designed to investigate knowledge sharing practices among African health sciences librarians. The specific research objectives are as follows:

1. To determine respondents' understanding of knowledge sharing
2. To discover types of knowledge shared among health science librarians in Africa
3. To establish the frequency and channels used for knowledge sharing among health science librarians in Africa
4. To identify the purposes of knowledge sharing among health science librarians in Africa
5. To ascertain the perceived benefits of knowledge sharing among health science librarians in Africa
6. To identify challenges to knowledge sharing among health science librarians in Africa

## METHODS

### Study design

A cross-sectional survey design study was adopted. The study population was 109 African health sciences librarians who attended the 16^th^ Biennial Conference of the Association for Health Information and Libraries in Africa (AHILA) held October 14–18, 2019, at the University of Ibadan in Nigeria. Delegates attending the conference included individuals from countries in Africa, Europe, and North America. However, the target population of the study was only delegates from African countries.

### Instrument

A structured questionnaire adapted from previous studies [10, 11, 13] was used to elicit responses from delegates. The face and context validity of the questionnaire was confirmed by senior colleagues and researchers with several years of experience in health librarianship. Cronbach's alpha was 0.72, indicating an acceptable level of internal consistency and reliability of the questionnaire.

### Study procedure

Copies of the questionnaire were handed out by the researcher to the target population and collected before the end of the conference. A total of ninety-one questionnaires were distributed to delegates who indicated willingness to participate in the study, out of which fifty-three were completed and used for analysis, resulting in a return rate of 58%. Collected data were analyzed using descriptive statistics with SPSS 21.0.

## RESULTS

### Respondent demographics

Respondents were asked about their job status, type of library, gender, age, academic qualifications, and years of professional working experience. Of the 53 respondents, 13 (24.5%) were head of libraries; 11 (20.8%) were senior librarian; 9 (17.0%) were librarian II; 9 (17.0%) were librarian I; 4 (7.5%) were assistant librarian; 2 (3.8%) were deputy librarian; and 5 (9.4%) were chief library officer, principal library officer, program manager, or lecturer. Twenty-four respondents (45.3%) came from academic libraries; 10 (18.8%) were from ministry of health, college of health science, school of nursing; 9 (17.0%) from teaching hospitals; 8 (15.1%) from biomedical research centers/institutes; and 2 (3.8%) from specialist hospitals. Thirty-three (62.3%) respondents were women; 20 (37.7 %) were men. Majority of the respondents 22 (41.5%) were 46–55 years of age; 14 (26.4%) were 36–45 years; 9 (17.0%) were older than 55 years; 5 (9.4%) were 25–35years; and 3 (5.7%) were below 25 years. Twenty-six (49.1%) respondents had a master's degree in library and information science (MLIS) or equivalent; 17 (32.1%) had a PhD; 6 (11.3%) had a BLIS/BA or equivalent; and 4 (7.5%) had other types of qualifications (i.e., diploma, postgraduate diploma, professional certificate). Fourteen (26.4%) respondents had been working professionally for 6–10 years; 11 (20.8%) for 11–15 years; 8 (15.1%) for 5 or fewer years; and 7 (13.2%) for 16–20 years. Finally, respondents came from 22 African countries, including Kenya, Cameroon, Mali, Nigeria, Congo Brazzaville, Seychelles, Niger, Ghana, Zambia, Zimbabwe, Mozambique, Malawi, Côte d'Ivoire, Senegal, Uganda, Botswana, South Africa, Guinea Bissau, Burkina Faso, Republic of Benin, Chad, and Tanzania.

### Respondents' understanding of knowledge sharing

Respondents had different perspectives of knowledge sharing, with 20.8% understanding it as communication of knowledge, 37.7% as exchange of knowledge, 11.3% as transmission and absorption of knowledge, and 64.2% as a combination of these three options.

### Types of knowledge shared

The main types of knowledge shared by respondents concerned conferences, workshops, and seminars (54.7%); new trends and technologies in librarianship (50.9%); knowledge newly learned by the respondent (35.8%); and scholarship availability (32.1%).

### Frequency of knowledge sharing

**Figure 1:**
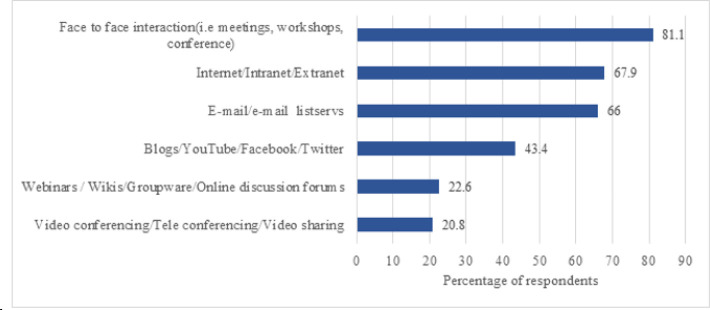
Channels of knowledge sharing among health sciences librarians in Africa

Respondents reported sharing their knowledge occasionally (35.8%), daily (34.0%), several times a month (13.2%), monthly (9.4%), or (7.5%) weekly. The most frequent channels of knowledge sharing were face-to-face interaction (e.g., meetings, workshops, conferences); Internet, intranet, or extranet; email or email listservs; blogs, YouTube, Facebook, or Twitter; webinars, wikis, groupware, or online discussion forums; and video conferencing, teleconferencing, or video sharing (Figure 1).

### Purposes for knowledge sharing

**Figure 2:**
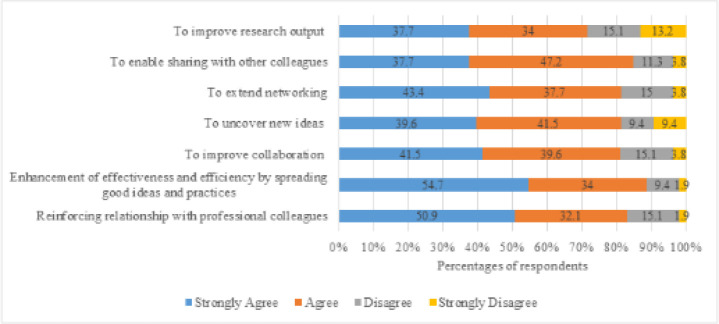
Purposes of knowledge sharing among health sciences librarians in Africa.

Most respondents strongly agreed that the purpose behind knowledge sharing is the enhancement of effectiveness and efficiency by spreading good ideas and practices (Figure 2). Many also strongly agreed that knowledge sharing reinforces relationships with professional colleagues, extends networking, improves collaboration, uncovers new ideas, enables sharing with other colleagues, and improves research output.

### Perceived benefits of knowledge sharing

**Figure 3:**
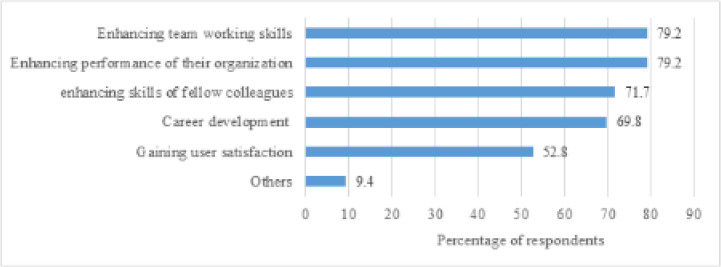
Benefits of knowledge sharing among health sciences librarians in Africa.

The main perceived benefits of knowledge sharing were enhancing team working skills, enhancing performance of their organization, enhancing skills of fellow colleagues, and career development (Figure 3).

### Barriers to knowledge sharing

**Figure 4:**
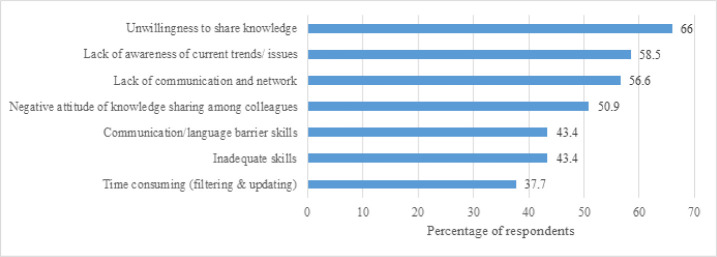
Barriers to knowledge sharing among health sciences librarians in Africa

The most frequently reported barriers to knowledge sharing were unwillingness to share knowledge, lack of awareness of current trends or issues, and lack of communication and networking (Figure 4).

## DISCUSSION

We found that health sciences librarians in Africa have different understandings of knowledge sharing, but most perceived knowledge sharing as a combination of communication of knowledge, exchange of knowledge, and transmission and absorption of knowledge. This result is consistent with the finding of Wei and colleagues [16] that knowledge sharing is the dissemination (i.e., communication) or exchange of knowledge, ideas, experiences, or skills transferred from one individual to another. A positive perception of knowledge sharing can serve as an impetus to share knowledge with the understanding that it could improve job performance, work quality, and task efficiency. With recent developments and new trends in the digital information environment, the cry for knowledge sharing and collaboration among health sciences libraries has grown even louder, leading many librarians to create forums for knowledge creation and dissemination [1]. Accordingly, for individuals to cultivate a knowledge sharing culture, they must have a positive perspective aimed at benefitting others.

As knowledge must be shared in order to make an impact, information professionals must believe that their knowledge, experience, or information received is valuable and worth sharing. We found that the main types of knowledge shared by African health sciences librarians were information on conferences, workshops, and seminars as well as information on new trends and technologies in librarianship. Conferences and workshops are veritable avenues for health sciences librarians to acquire new knowledge and enhance their professional skills; thus, knowledge sharing about such professional meetings has become utilitarian. This finding indicates that knowledge sharing exists among health science librarians in Africa, who recognize the need for networking and professional collaboration with colleagues in the same field who are likewise seeking to acquire new knowledge and skills and to keep pace with current trends in their area of practice. This outcome corroborates the finding of Akparobore that librarians prefer to share knowledge about ICT/networking, knowledge management, and issues that would benefit their professional colleagues [6]. In this context, librarians are not only information providers but also knowledge seekers. Hence, the type and extent of information received from colleagues would not only improve one's level of awareness in specialized areas but also increase one's skills and capacities.

The channels used for knowledge sharing can play an important role in individuals' willingness to share knowledge and the extent to which large audiences can be reached. We found that the main channel used for knowledge sharing among health sciences librarians in Africa was face-to-face interaction. This may explain the reason why we also found that most respondents shared knowledge only occasionally. This outcome is consistent with the finding of Ogunmodede and Popoola that the main channel used was face-to-face interaction [10]. However, this suggests that many respondents have not fully adopted the use of modern technologies and social media for knowledge sharing. This outcome is contrary to the finding of Quadri and Garaba that most respondents used ICT, as it helps them quickly deliver and disseminate knowledge [11]. There has generally been universal recognition and preference for ICT use for knowledge sharing, as it helps bridge the digital gap created as a result of physical distance. The advancement of ICT, especially social media, brings new opportunities for knowledge sharing and networking as it allows members of the global community to easily reach one and another. It is likely that the few health sciences librarians in Africa, who engage in high-level knowledge sharing, using new tools and technologies would influence others positively and in a more effective manner. However, it must be noted that the degree of ICT penetration in developing countries still differs, meaning that accessibility and usability still remain big challenges. Although libraries and librarians in low-income countries may be slower to adopt new practices, it is obvious that some librarians have accepted this challenge and are assuming new roles.

Knowledge sharing is increasingly encouraged due to its perceived benefits [7]. We found that the main motives behind knowledge sharing among health sciences librarians in Africa are the enhancement of effectiveness and efficiency by spreading good ideas and practices and the reinforcement of relationships with professional colleagues. This outcome further supports the tenet of SET that professional colleagues tend to build social relationships with others by sharing their knowledge, experience, and skills through a sense of responsibility to help others within their enclave [6]. The more knowledge is shared, the more knowledge is generated, hence resulting in further innovation and organizational success. Therefore, knowledge sharing should be at the heart of every librarian, considering the benefits that can be derived. Many libraries encourage their staff to participate in knowledge sharing and professional networking given that it provides opportunities for them to actively engage with staff within other libraries and to tap into activities beyond their own environment. This is in agreement with Mayekiso [9], who found that knowledge sharing enables librarians to stay abreast of new developments in their profession and enhances the efficiency and efficacy of service delivery.

Furthermore, we found that health sciences librarians in Africa face challenges to knowledge sharing, with the top challenge being unwillingness to share knowledge. As willingness to share knowledge, experiences, and skills is a voluntary action, the more health sciences librarians recognize the importance of knowledge sharing as a social norm, the more they will enthusiastically share. Unless individuals share knowledge they gain, that knowledge may have only a limited impact. The reasons for unwillingness to share knowledge may include poor individual understanding, lack of skills in using technological tools, and inadequate motivation. Other challenges identified by respondents were a lack of awareness of current trends/issues, lack of communication and networking, negative attitude about knowledge sharing among colleagues, and communication/language barriers. This finding supports earlier findings of Fari and Ocholla as well as Awodoyin and colleagues, who identified many of the same factors hindering knowledge sharing among their study respondents [13, 14]. Despite these barriers, it is imperative for librarians to find means of sharing knowledge with their colleagues.

Based on the outcomes of this study, we recommend that AHILA endeavors to conduct training for both long-standing and new members to further improve their awareness of the importance of knowledge sharing. Since the use of technology, especially social media, is recognized as an effective and easy medium for sharing knowledge [12], there is a need for health sciences librarians in Africa to acquaint themselves with new technologies. Finally, challenges to knowledge sharing among health sciences librarian in various African countries should be further studied and concerted efforts should be made to address them.

A limitation to this study is its small sample size, as not all delegates filled out and returned their questionnaire. Also, as characteristics (e.g., awareness, skills) and environments (e.g., country) differed among respondents, generalizing the findings should be done cautiously. Despite these limitations, our study contributes to knowledge sharing among health sciences librarians in Africa, which has not been previously researched to any extensive degree. We hope that this study helps increase awareness of the importance and benefits of knowledge sharing among health sciences librarians.

## Data Availability

Data underlying our results are available via the Open Science Framework at https://osf.io/aw93q/.

## SUPPLEMENTAL FILES

Survey Questionnaire
