# Supplementary material for: Knowledge sharing practices among African health sciences librarians
Source: J Med Libr Assoc. 2021 Oct 1;109(4):624–30. doi: 10.5195/jmla.2021.1183 (PMC8608209; doi:10.5195/jmla.2021.1183)
Supplement: Supplementary file 1 — Survey Questionnaire [file jmla-109-4-624-s01.docx]

**Survey Questionnaire**

**Dear Professional Colleagues,**

I kindly request you to participate in this survey that aims at assessing different issues on Knowledge sharing practices among African Health Sciences Librarian. Participation is purely optional. Please tick [√] the answers from the options mentioned. Your responses will be treated with confidentiality.

Thank you in anticipation for sparing a part of your valuable time to participate in the survey.

**Consent**

I voluntary consent to my participation in this study.

Name………………………………..

Signature of participant……………………

***Section A: Demographic Information***

1. **Category of Librarian:** Head of Library [ ] Deputy Librarian [ ] Senior Librarian [ ] Librarian 1 [ ] Librarian11 [ ] Ass. Librarian [ ] Others……………..
2. **Type of Health Institution you work:** Teaching hospital [ ] Academic library [ ] Biomedical Research Center/Institute [ ] Specialist Hospital [ ] Others (specify)……………………….
3. **Gender of the Respondent**: Male [ ] Female [ ]
4. **Age range**: Below 25[ ] 25–35years [ ] 36–45 years [ ] 46–55 years[ ] 55 years & above[ ]
5. **Highest Educational Qualification:** BLIS/BA or Equivalent Degree [ ] MA / MLIS or Equivalent Degree [ ] PhD [ ] Others (specify)……………
6. **Professional Experience:** < 5 [ ] 6-10 [ ] 11-15 [ ] 16-20 [ ] More than 20 [ ]
7. **Country**:………………………

***Section B: Knowledge Sharing***

1. **What is your understanding of knowledge sharing?** *Please express your view from the following options*.
2. Communication of knowledge [ ]
3. Exchange of knowledge [ ]
4. Transmission and absorption of knowledge [ ]
5. Combination of these three options [ ]
6. **Kindly indicate the types of knowledge you shared with your professional colleagues**

| **Statement** | **Very much** | **Much** | **Less** | **Never** |
| --- | --- | --- | --- | --- |
| Information on conferences, workshops and seminars |  |  |  |  |
| Information on scholarship availability |  |  |  |  |
| Information on new trends / technologies in librarianship |  |  |  |  |
| Sometimes, I shared information of new knowledge learned |  |  |  |  |

1. **Kindly indicate the frequency of your knowledge sharing among your professional colleagues**
2. Daily [ ] b. Weekly [ ] c. Monthly [ ] d. Occasionally [ ] d. Several times a month [ ]
3. **Kindly indicate channels used for knowledge sharing among your professional colleagues (***Please express your view from the following options)*.

| **Channels** | ***Tick*** |
| --- | --- |
| Internet/Intranet/Extranet |  |
| E-mail/ E-mail listservs |  |
| Through face to face interaction(i.e meetings, workshops, conference) |  |
| Video conferencing/Tele conferencing/Video sharing |  |
| Blogs/YouTube/Facebook/Twitter |  |
| Webinars / Wikis/Groupware/Online discussion forums |  |
| Others (specify) |  |

1. **Purpose for information and knowledge sharing**

| **Purpose** | **Strongly Agree** | **Agree** | **Disagree** | **Strongly Disagree** |
| --- | --- | --- | --- | --- |
| Reinforcing relationship with professional colleagues |  |  |  |  |
| Enhancement of effectiveness and efficiency by spreading good ideas and practices |  |  |  |  |
| To improve collaboration |  |  |  |  |
| To uncover new ideas |  |  |  |  |
| To extend networking |  |  |  |  |
| To enable sharing with other colleagues |  |  |  |  |
| To improve research output |  |  |  |  |

1. **What are your perceived benefits of knowledge sharing?** *Please express your view from the following options*.

| **Benefits** | ***Tick*** |
| --- | --- |
| Enhancing skills of fellow colleagues |  |
| Enhancing team working skills |  |
| Enhancing performance of the organization |  |
| Gaining user satisfaction |  |
| Career development |  |
| Others ………………….. |  |

1. **What are the challenges militating against your knowledge sharing?** *Please express your view from the following options*.

| **Barriers** | ***Tick*** |
| --- | --- |
| Negative attitude of knowledge sharing among colleagues |  |
| Unwillingness to share knowledge |  |
| Time consuming (filtering & updating) |  |
| Lack of awareness of current trends/ issues |  |
| Lack of communication and network |  |
| Inadequate skills |  |
| Communication/language barrier skills |  |
